# Incorporating physiologically relevant mobile phases in micellar liquid chromatography for the prediction of human intestinal absorption

**DOI:** 10.1002/bmc.4351

**Published:** 2018-08-16

**Authors:** Dina S. Shokry, Laura J. Waters, Gareth M. B. Parkes, John C. Mitchell

**Affiliations:** ^1^ Faculty of Engineering and Science, Medway Centre for Formulation Science University of Greenwich Kent UK; ^2^ School of Applied Sciences University of Huddersfield Huddersfield UK

**Keywords:** bile salt, HIA, lipophilicity, MLC, *P*_mw_

## Abstract

Micellar liquid chromatography is a popular method used in the determination of a compound's lipophilicity. This study describes the use of the obtained micelle–water partition coefficient (log *P*_mw_) by such a method in the prediction of human intestinal absorption (HIA). As a result of the close resemblance of the novel composition of the micellar mobile phase to that of physiological intestinal fluid, prediction was deemed to be highly successful. The unique micellar mobile phase consisted of a mixed micellar mixture of lecithin and six bile salts, i.e. a composition matching that found in the human intestinal environment, prepared in ratios resembling those in the intestine. This is considered to be the first method to use a physiological mixture of biosurfactants in the prediction of HIA. As a result, a mathematical model with high predictive ability (*R* ^2^_PRED_ = 81%) was obtained using multiple linear regression. The micelle–water partition coefficient (log *P*_mw_) obtained from micellar liquid chromatography was found to be a successful tool for prediction where the final optimum model included log *P*_mw_ and polar surface area as key descriptors with high statistical significance for the prediction of HIA. This can be attributed to the nature of the mobile phase used in this study which contains the lecithin–bile salt complex, thus forming a bilayer system and therefore mimicking absorption across the intestinal membrane.

**Abbreviations**
GC: sodium glycocholate
GDC: sodium glycodeoxycholate
HIA: human intestinal absorption
MLC: micellar liquid chromatography
NaDC: sodium deoxycholate
NaTC: sodium taurocholate
NaTDC: sodium taurodeoxycholate
PSA: polar surface area.

## 1 INTRODUCTION

The oral route is the most popular route of administration for pharmaceutical entities. However, the properties of compounds must be suited to delivery via this route (Arlington, 2000; Kennedy, 1997; Prentis, Lis, & Walker, 1988; Venkatesh & Lipper, 2000). Early identification of drug candidates with poor biopharmaceutical properties, such as poor aqueous solubility and oral bioavailability, is advantageous to avoid potential economic loss on subsequent unsuccessful clinical research. As a result, there has been a growing interest in the early prediction of biopharmaceutical properties by means of experimental and theoretical models.

Drug solubility and permeation are the two main properties that affect drug absorption from the intestinal lumen (Amidon, Lennernäs, Shah, & Crison, 1995; Johnson & Swindell, 1996; Norris, Leesman, Sinko, & Grass, 2000). Once identified, drugs with poor solubility have a greater possibility for improvement when compared with those with low intestinal permeability as drug solubility can be enhanced by choosing a more suitable formulation option. As a consequence of this, focussed synthesis of compounds with structures of reasonably high permeability during the early stages of drug development is preferential. Since drug lipophilicity is considered a key descriptor that dictates permeation across biological membranes (Rutkowska, PajIk, & Jóźwiak, 2012), the evaluation or determination of the lipophilicity of a drug is important for its characterization to ensure its potential to penetrate lipid barriers and subsequently be absorbed (Lipinski, Lombardo, Dominy, & Feeney, 2001; Scott & Clymer, 2002). Therefore, determining the lipophilicity of a compound can help in the prediction of human intestinal absorption (HIA).

Having the ability to explore the effects of micelles on the behaviour of a compound, micellar liquid chromatography (MLC) has been developed over the past 30 years to yield information on a wide variety of compounds where a surfactant aqueous solution is used above its critical micellar concentration (CMC; Berthod & Garcia‐Alvarez‐Coque, 2000) (Ruiz‐Ángel, García‐Álvarez‐Coque, & Berthod, 2009). A very important physicochemical property indicating lipophilicity, log *P*_mw_, can be obtained using MLC in the presence of different surfactants as the micellar mobile phase to help characterize compounds (Kawczak et al., 2010; Marina & Garcia, 2000). For example, MLC has been used with a simple surfactant solution in previously published work for the prediction of HIA for a series of compounds using multiple linear regression analysis (Waters, Shokry, & Parkes, 2016). The novelty of the work presented in this study lies in the use of a very unique mobile phase mimicking the *in vivo* intestinal environment of humans. The composition of this mobile phase was closely related to that of the intestinal fluid through a combination of lecithin and bile salts which are normally found in the human intestine, used in ratios matching those found physiologically (Wiedmann, Liang, & Kamel, 2002).

## 2 METHODS AND MATERIALS

Sodium deoxycholate (NaDC; 97%), sodium taurodeoxycholate (NaTDC; 95%), sodium taurocholate (NaTC; ≥97%), sodium cholate (NaC; 97%), sodium glycocholate (GC; ≥97%), sodium glycodeoxycholate (GDC; ≥97%) and l‐*α*‐phosphatidylcholine from dried egg yolk (≥50%) were used as purchased from Sigma Aldrich, Dorset, UK for the preparation of stock solutions of mobile phase. Analytical grade 4‐(2‐hydroxyethyl)‐1‐piperazineethanesulfonic acid (HEPES buffer) was purchased from Sigma Aldrich, Dorset, UK. The compounds considered in this work were caffeine 97% (Sigma Aldrich, Dorset, UK), fenoprofen 97% (Fluka, Dorset, UK), acetaminophen 99% (Sigma Aldrich, Dorset, UK), ketoprofen 98% (Sigma Aldrich, Dorset, UK), phenylbutazone 99% (Sigma Aldrich, Dorset, UK), fluconazole 98% (Sigma Aldrich, Dorset, UK), carbamazepine 99% (Sigma Aldrich, Dorset, UK), cimetidine (Sigma Aldrich, Dorset, UK), naproxen 98% (Sigma Aldrich, Dorset, UK), terbutaline 96% (Sigma Aldrich, Dorset, UK), zolmitriptan >98% (Sigma Aldrich, Dorset, UK), salicylic acid 99% (Fisher Scientific, Loughborough, UK), ibuprofen 98% (BASF, Cheshire, UK), acetyl salicylic acid 99% (Acros Organics, Geel, Belgium), diclofenac 98% (TCI Europe, Zwijndrecht, Belgium), flurbiprofen 98% (TCI Europe), nicotinic acid >98% (Sigma Aldrich, Dorset, UK) and theophylline 98%, (TCI, Oxford, UK).

### 2.1 Preparation of stock solution of micellar mixture simulating the physiological bile salt mixture

A 17 mM stock solution of a mixed micellar system was prepared by transferring accurately weighed amounts equivalent to 2.71, 2.00, 2.08, 2.08, 4.70 and 3.43 mM of NaTC, NaTDC, NaDC, NaC, NaGC and NaGDC bile salts respectively and 0.75 mM of egg phosphatidylcholine to a 250 mL volumetric flask with buffer solution (10 mM HEPES, pH 6.5) in 0.15 M NaCl. The solution was then sonicated for 30 min and stored for 12 h before use to allow the formation of stable mixed micelles.

### 2.2 Preparation of a mixed micellar solution for dilution

Different concentrations of the micellar mixture were prepared over the range of 5–17 mM by diluting the stock solution using a 2 mM mixture solution. The 2 mM mixture solution contained the same six bile salts and lecithin used in the preparation of the stock mixture solution in the same molar ratios. The 2 mM diluting mixture was prepared by transferring accurately weighed amounts equivalent to 0.32, 0.25, 0.24, 0.24, 0.55 and 0.4 mM of NaTC, NaTDC, NaDC, NaC, NaGC and NaGDC bile salts, respectively, and 0.75 mM of egg phosphatidylcholine to a 250 mL volumetric flask with buffer as detailed previously. The resultant solution was sonicated for 30 min then stored for 12 h before use. Dilution was carried out in this way as the 2 mM mixture is considered to be the monomer bile salt concentration that is required to be kept constant in each solution in order to keep the size of the micelle constant while its concentration is being changed (Wiedmann et al., 2002).

### 2.3 Analytical instrumentation and measurement

Experiments were carried out with a chromatographic system consisting of an Agilent 1100 series binary pump, a Rheodyne injector through which 20 μL samples were injected in to the system and a UV detector (Perseptive Biosystems UVIS‐205), set at a wavelength appropriate for each drug producing a peak via Picolog software indicating the retention of the solute within the column as a function of time. The mobile phase was filtered through a 0.45 μm Nylon filter and degassed in an ultrasonic bath. Data were recorded and then analysed to obtain retention factors and each run was repeated three times to ensure that reasonable accuracy and precision were achieved. Analytical separation was accomplished using a reversed phase cyanopropyl column (Spherisorb 5 μm, 15 cm × 4.6 mm i.d., Waters). The flow rate used was 1.34 mL/min with all assays carried out at 37°C. The mobile phase was placed in a water bath at 37°C throughout the duration of all experiments.

### 2.4 Determination of dead time *t*_0_

The dead time (*t*_0_) is defined as the time taken by the solvent front to reach the detector, measured by the injection of water (Pramauro, Minero, Saini, Graglia, & Pelizzetti, 1988) or an organic solvent, e.g. acetonitrile or methanol (Khaledi, 1988; Khaledi, Peuler, & Ngeh‐Ngwainbi, 1987). In this work, dead time was determined by injecting distilled water or acetonitrile in to the system and recording the retention time of the first peak that appeared after injection (solvent front). The same method was repeated for each of the bile salt concentrations used. A reliable value of dead time used in the calculation of retention factor (*k*′) for all experiments (using eqn (1)) was determined from an average of at least 10 recordings.

### 2.5 Calculation of log *P*_mw_

Retention behaviour of binding solutes as a function of the micellar concentration [*M*] (concentration of surfactant monomers forming micelles equal to total surfactant concentration minus the CMC) has been explained by many proposed theoretical approaches such as the Armstrong and Nome partitioning model, the Arunyanarat and Cline‐Love model and the Foley model (Armstrong & Nome, 1981; Arunyanart & Love, 1984; Garcia‐Alvarez‐Coque, Torres‐Lapasió, & Baeza‐Baeza, 1997).

**Figure 1:**
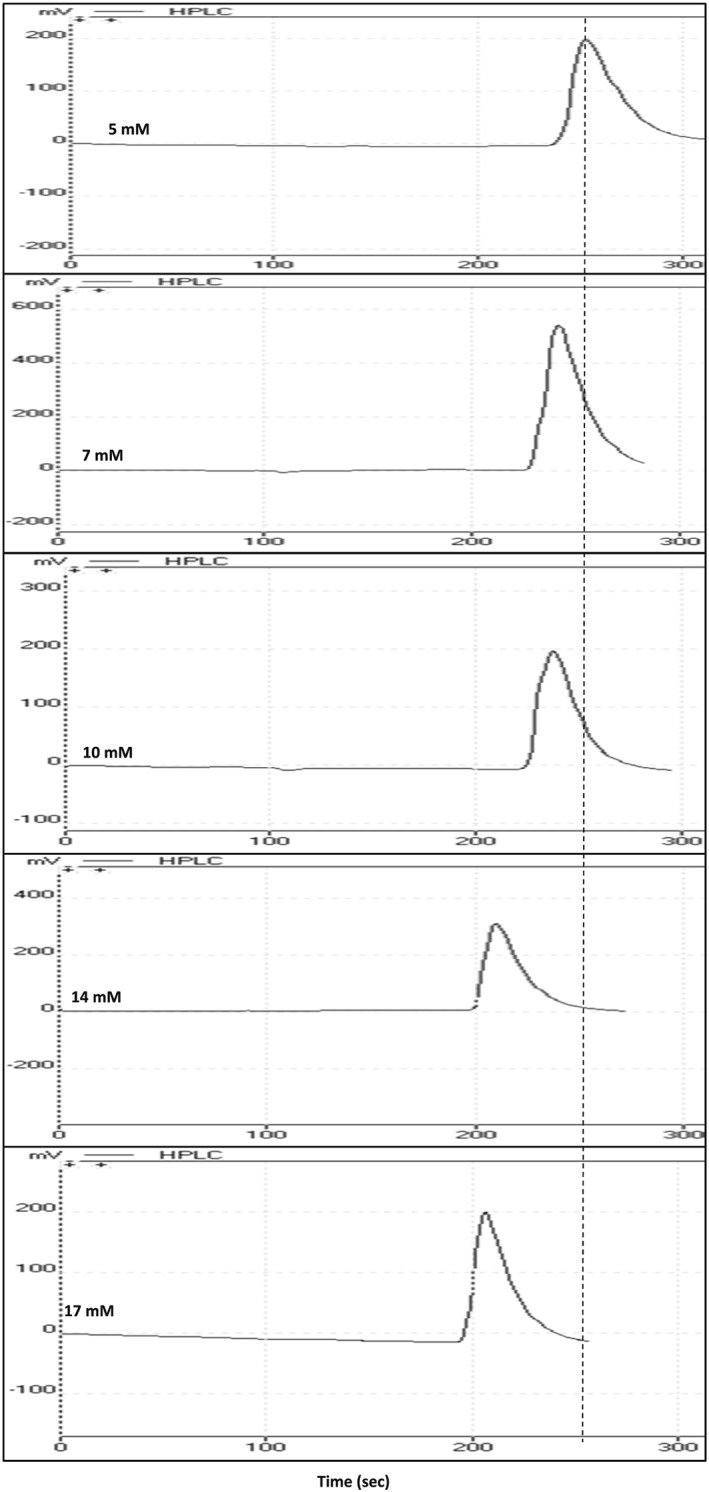
Chromatograms showing binding behaviour of ketoprofen in increasing concentrations of physiological micellar bile salts mixture as a mobile phase (the dotted line is only used for visual guidance)

From the obtained chromatograms, the retention time of each drug was recorded for each bile salt concentration (Figure 1). The retention factor for each retention time was calculated using the following equation:

(1) $$k\prime =\frac{\left(\text{Retention time}-\text{dead time}\right)\;}{\text{dead time}}$$

The reciprocal of each retention factor was obtained (1/*k*^′^) with the average value plotted against the micellar concentration (*C*_M_) that was calculated according to the following equation:

(2) $$\left(C_{M}\right)=\text{total surfactant concentration}-\mathrm{CMC}\;$$

The partition coefficient (log *P*_mw_) was obtained from the slope and intercept of the line obtained from the plot of (*C*_M_) against (1/*k*^′^):

(3) $$\log\;P_{\mathrm{mw}}=\log\left[\text{intercept}/\text{slope}\right]$$

## 3 RESULTS

### 3.1 Mixed micellar system

Since bile salts and lecithin (phosphatidylcholine) are considered to be two of the most common biosurfactants present in bile and involved in the digestion process, it was important to study the effect of using a mixed micellar system consisting of six bile salts and lecithin phospholipid as a mobile phase in MLC. The mixed micellar system used in this method consisted of a mixture of six bile salts (NaDC, NaC, NaTDC, NaTC, NaGC and NaGDC) which included dihydroxy, trihydroxy, conjugated and unconjugated bile salts with lecithin phospholipid in 0.15 M NaCl with the pH controlled by HEPES buffer at 6.5. The CMC of the mixed micellar system was deemed to be 0.0046 M based on the average value of the CMCs of the bile salts included in the mixture in 0.15 M NaCl [NaTC CMC = 0.004 M (Natalini et al., 2014), NaDC CMC = 0.0024 M (Natalini et al., 2014), NaTDC CMC = 0.0024 M (Natalini et al., 2014), NaC CMC = 0.0075 M (Reis et al., 2004), NaGC CMC = 0.009 M (Natalini et al., 2014) and NaGDC CMC = 0.0022 M (Natalini et al., 2014)]. The bile salt–lecithin mixed micellar solution was used over a concentration range 0.005–0.017 M. The mixed micellar system was prepared in molar ratios similar to that present physiologically (Wiedmann et al., 2002).

Having both a positively charged choline head group and a negatively charged phosphate group, lecithin is considered to be a zwitterionic compound that tends to self‐assemble in water, forming characteristic bilayer membrane‐like structures (Cheng, Oh, Wang, Raghavan, & Tung, 2014). Bile salts are distinguishable from conventional amphiphiles by their facial structure with polar and nonpolar faces. Such uniqueness is what leads to the unusual micelle structures formed upon bile salts’ self‐assembly in water, which further separates them from conventional head and tail surfactants. Various models have been proposed for bile salt micelle formation and several hypotheses have been made regarding their aggregates’ structures formed through hydrophobic interactions between the steroid nuclei of bile salts (nonpolar face) and the hydrogen bonding between the bile salts hydroxyl groups (polar face) (Malik, 2016). It was reported in previous studies that short, rod‐like micelles were formed upon combining both bile salts and lecithin in a mixture (Cheng et al., 2014). The lecithin–bile salt complex is considered as a balanced system where the lecithin on its own in water forms unstable bilayer structures of low aqueous solubility because of its bulky hydrophobic tails inhibiting its solubility in water that is compensated for and balanced by the presence of the bile salts of much greater water solubility. These can, in small amounts, stabilize the lecithin self‐assembled structures by intercalating into these structures and thus promoting their water solubility, which is one of the main physiological applications of bile salts.

Initially, it was suggested by Mazer, Benedek and Carey that the aqueous lecithin/bile salt micelles were disc‐like in shape (Mazer, Benedek, & Carey, 1980) but later on, different techniques provided evidence that these micelles are cylindrical in shape and can further grow into long flexible cylindrical micellar chains termed “worms” (Madenci, Salonen, Schurtenberger, Pedersen, & Egelhaaf, 2011; Walter, Vinson, Kaplun, & Talmon, 1991) which are similar to polymer chains where they entangle in a transient network rendering the solution highly viscous (Dreiss, 2007; Schurtenberger, Scartazzini, & Luisi, 1989; Shchipunov, 2001). This transformation of short cylinders to worms depends on the molar ratio of the two species and the ionic strength where an almost equimolar ratio of bile salt–lecithin (with high background counterion concentration) would induce the growth of the cylindrical micelles to worms (Cheng et al., 2014). As a result, caution was taken to avoid the formation of a highly viscous solution since the prepared micellar mixture was to be pumped through the chromatographic system. For this reason, the bile salt–lecithin mixed micellar system was prepared in a molar ratio much higher than that using an optimum counterion concentration (0.15 M NaCl).

**Figure 2:**
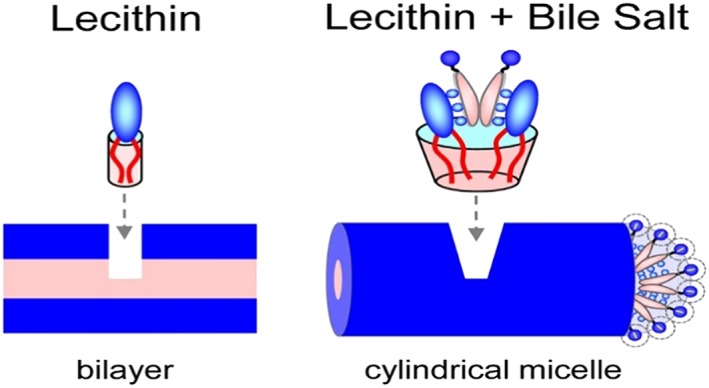
Schematic of the self‐assembled structures formed by lecithin with, and without, bile salt in water (Cheng et al., 2014)

Lecithin prefers to be present in the form of low‐curvature cylindrical‐shaped bodies owing to its two tails. It is expected that the bile salts will stabilize the hemispherical end caps of these cylinders as bile salts are generally present in water as highly curved small micelles. Since stable end caps prevent the formed cylindrical micelles from further growing into long chains, adding more bile salts will result in more end caps being formed and therefore shorter cylinders (Cheng et al., 2014). Figure 2 summarizes the mechanism of micellization in the bile salt–lecithin mixed micellar system where lecithin prefers to form bilayers when alone in water (left side of the figure). On the other hand, when bile salts are added to the solution they bind to lecithin head groups (Cheng et al., 2014) with themselves binding back‐to‐back (Coello, Meijide, Núñez, & Tato, 1996) to each other, resulting in expansion of the head group area (right side of the figure) (Cheng et al., 2014; Madenci et al., 2011). As a result, bilayers turn into cylinders where the net geometry changes from a cylinder to truncated cone. In the case of low ionic strength, the negatively charged groups of bile salts suffer from high repulsion forces; therefore bile salts get packed at the curved hemispherical end caps of the cylinders. The presence of a counterion (NaCl) of an optimum concentration is important because it decreases or neutralizes the surface charge on the micelle, thereby diminishing electrostatic repulsion and encouraging interaction between micelle‐forming species and hydrophobic association of bile salts and lecithin to give mixed micelles. It has to be taken into consideration that, upon increasing the concentration of counterion, the electrostatic repulsion between the bile salts decreases; therefore the aggregation number of bile salt micelles increases and bile salts become less likely to form the highly curved end caps of the cylindrical mixed micelles, inducing the growth of cylinders in to long chains which increase the viscosity of solution.

### 3.2 Retention behaviour

Ideally when using an anionic surfactant and a cyanopropyl column, neutral and cationic drugs are expected to show a binding interaction as a result of entrapment of drug in the hydrophobic core of the micelles (for neutral drugs) or electrostatic attraction (for cationic drugs) or both, which leads to a decrease in the retention times of these drugs with the increase in the mobile phase micellar concentration. On the other hand, anionic drugs are expected to show an antibinding interaction owing to electrostatic repulsion between the anionic drug molecules and the anionic micelles, which leads to binding of these drug molecules to the cyanopropyl column, increasing their retention times with the increase in the micellar concentration in the mobile phase (Armstrong & Stine, 1983; Ruiz‐Angel, Carda‐Broch, Torres‐Lapasió, & García‐Álvarez‐Coque, 2009).

However, all the drug molecules in this study (neutral, cationic and anionic) show a binding behaviour reflecting the preference of the analysed drugs to the bile salt–lecithin mixed micelles of more stability, bigger hydrophobic core diameter and core fluidity (de Castro, Gameiro, Guimarães, Lima, & Reis, 2001). The binding of anionic drug molecules to these mixed micelles could be attributed to diminished repulsion forces between the micelles resulting from charge neutralization brought about by counterion (NaCl) binding, therefore overcoming any remaining weak repulsion forces and not repelling away from the micelles, i.e. solubilizing within the hydrophobic core of the mixed micelles.

#### 3.2.1 Statistical modelling of HIA

Following analysis of a group of 18 model drugs using a physiologically simulating bile salt–lecithin mixed micellar solution, followed by calculation of log *P*_mw_ from the calibration plots of 1/*k*^′^ against *C*_M_, the obtained log *P*_mw_ and a number of other molecular descriptors such as molecular weight, polar surface area (PSA), freely rotating bonds, molar volume, dissociation constant (p*K*_a_), aqueous solubility, number of hydrogen bond donors and number of hydrogen bond acceptors were used for developing a model for prediction of %HIA. Experimentally obtained log *P*_mw_ values (using this MLC method) with PSA as the selected molecular descriptor included in the final model are shown in Table 1.

**Table 1:** Calculated log *P*_mw_ values (using experimental micellar liquid chromatography, MLC, data) and literature values of polar surface area (PSA) for the 18 model compounds (https://pubchem.ncbi.nlm.nih.gov/, December 2014)

Drug	Log *P*_mw_	PSA
Acetaminophen	1.31	49.3
Acetylsalicylic acid	1.74	63.6
Caffeine	0.93	58.4
Carbamazepine	2.39	46.3
Cimetidine	1.97	88.89
Diclofenac	2.94	49.3
Fenoprofen	2.52	46.5
Fluconazole	1.40	81.6
Flurbiprofen	2.55	37.3
Ibuprofen	1.52	37.3
Ketoprofen	1.58	54.4
Naproxen	2.37	46.5
Nicotinic acid	1.55	50.2
Phenylbutazone	2.15	40.6
Salicylic acid	1.69	57.5
Terbutaline	2.96	72.7
Theophylline	1.02	69.3
Zolmitriptan	2.30	57.4

For improving the linear relationship found between reported %HIA and experimental log *P*_mw_ values, logit (HIA) was used as reported in similar studies (Norinder, Österberg, & Artursson, 1999; Raevsky, Fetisov, Trepalina, McFarland, & Schaper, 2000; Zhao et al., 2002). As a result, transformation of human intestinal absorption values to logit was carried out by substituting in eqn (4):

(4) $$\text{Logit}\;\left(\%\mathrm{HIA}\right)=\log\;\left(\%\mathrm{HIA}/\left(100-\%\mathrm{HIA}\right)\right)$$

Removal of drugs with %HIA values of 100 or 0% from the training set was essential.

Minitab 17^®^ was used in the statistical analysis of data. Data was analysed using multiple linear regression where all of the previously mentioned molecular descriptors (data not shown) were included and regressed against the dependant variable %HIA and backward elimination modelling strategy was used. As a result, PSA along with log *P*_mw_ were the only descriptors included in the final developed model. Variables with high variance inflation factors were removed to keep it within the acceptable limits. Finally, an optimum model was obtained that provided a good summary of data. Assessment of the variables remaining in the final model for significance and relative importance was carried out using standardized coefficients and the associated *p*‐values.

The final model predictive ability was evaluated using adjusted‐*R* ^2^ and *R* ^2^ for prediction (*R* ^2^_PRED_), which is able to indicate the predictive ability of the model and consequently reflecting the capability for applying the model.

Log *P*_mw_ was included in a model equation with %HIA experimental values for orally administered drugs which allowed the prediction of %HIA. The model obtained for the prediction of %HIA is given by eqn (5):

(5) $$\text{logit}\;\mathrm{HIA}=4.103–0.939\;\log\;P_{\mathrm{mw}}-0.02218\;\mathrm{PSA}$$

Fifteen drugs were used in the development of the final model where log *P*_mw_ alongside the molecular descriptor (PSA) was included. The model's *R* ^2^ = 86.40%, *R* ^2^_adjust._ = 84.13%, *R* ^2^_PRED_ = 80.73% and *S* = 0.247.

**Figure 3:**
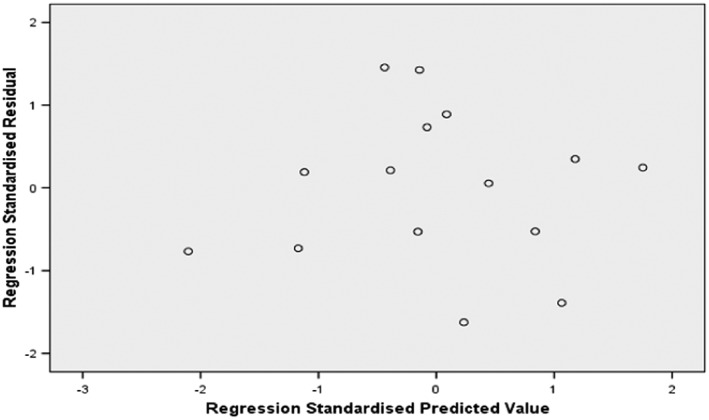
Residual plot for optimal logit human intestinal absorption (HIA) regression model

**Figure 4:**
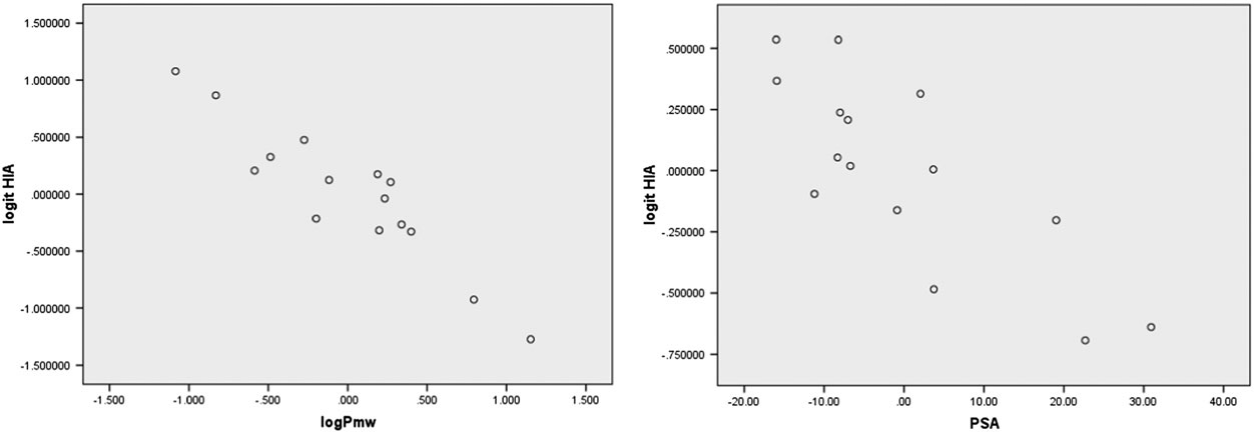
Partial regression plots of experimental logit HIA values against log *P*_mw_ and polar surface area (PSA)

**Figure 5:**
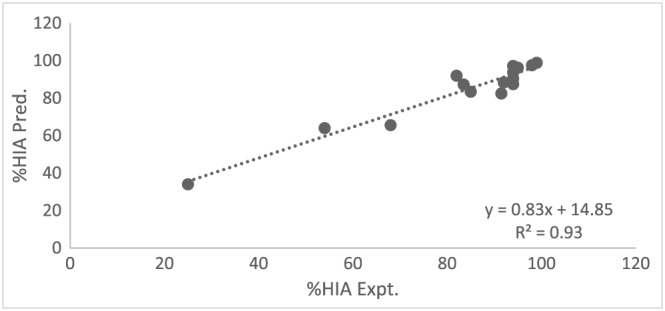
Regression plot of predicted %HIA values against the literature %HIA

A 95% confidence interval for log *P*_mw_ is given by (−1.18, −0.699). The *t*‐statistic and standardized coefficient of log *P*_mw_ are −8.51 (*p* < 0.05) and − 0.964, respectively, suggesting the statistical significance of log *P*_mw_ as a predictor. Also the *F*‐ratio of the overall model is statistically significant, *F* = 38.12 and the *p‐*value is 0.007 (*p* < 0.05). Figure 3 shows no marked relationship between residuals and predicted values while Figure 4 summarizes the model. The literature and predicted values of %HIA are shown in Table 2 and Figure 5. Three drugs (acetaminophen, ibuprofen and salicylic acid) were used to test the obtained model. The model was able to predict the %HIA for these compounds within a minimum of 0.61% and a maximum of 4.43% difference between predicted and published data for %HIA. The statistical model developed from this study using a bile salts–lecithin physiological mixture confirms an enhanced capability for prediction of HIA, with an *R* ^2^_PRED_ of 81%, compared with a previous study using simple micelles of one bile salt (*R* ^2^_PRED_ of 75%; Waters et al., 2016). The current model involves fewer predictors (log *P*_mw_ and polar surface area) than those in the previous model (log*P*_mw_, molecular weight and solubility), which simplifies the model. Based on previous research, PSA has been reported to be a successful parameter in the prediction of intestinal absorption (Clark, 1999; Palm, Stenberg, Luthman, & Artursson, 1997; Stenberg et al., 1999). Furthermore, drug absorption‐relevant information has been shown to be sufficiently encoded in lipophilicity, along with PSA, without explicit reference to molecular weight (Egan, Merz, & Baldwin, 2000). These findings further corroborate that the current model displays superiority over the previously developed one for predicting intestinal absorption.

**Table 2:** Experimental and predicted values for %HIA

Drug	Experimental. %HIA	Reference	Predicted %HIA
Acetaminophen*	100	(Castillo‐Garit, Cañizares‐Carmenate, Marrero‐Ponce, Abad, & Torrens (2014)	98
Acetylsalicylic acid	82	Castillo‐Garit et al. (2014)	92
Caffeine	99	Yan, Wang, & Cai (2008)	99
Carbamazepine	84	Dressman, Amidon, & Fleisher (1985); Varma, Sateesh, & Panchagnula (2005)	87
Cimetidine	68	Castillo‐Garit et al. (2014); Linnankoski, Mäkelä, Ranta, Urtti, & Yliperttula (2006)	66
Diclofenac	54	Veber et al. (2002)	64
Fenoprofen	85	Hou, Wang, Zhang, & Xu (2007)	83
Fluconazole	94	Newby, Freitas, & Ghafourian (2015)	90
Flurbiprofen	92	Raevsky (2004)	88
Ibuprofen*	98	Newby et al. (2015)	99
Ketoprofen	95	Newby et al. (2015)	96
Naproxen	94	Castillo‐Garit et al. (2014)	87
Nicotinic acid	94	Newby et al. (2015)	97
Phenylbutazone	94	Hou et al. (2007); Veber et al. (2002)	94
Salicylic acid*	99	Raevsky (2004)	95
Terbutaline	25	Grès et al. (1998)	34
Theophylline	98	Kansy, Senner, & Gubernator (1998)	98
Zolmitriptan	92	Newby et al. (2015)	82

* Validation compounds.

## 4 CONCLUSION

Development of an MLC method that used a physiologically resembling bile salt–lecithin mixed micellar system was successful for the prediction of HIA. This method had a significant impact on the elution of compounds and the type of interaction they experienced upon being injected into the MLC system. The bile salt–phospholipid combination had a higher solubilizing capacity for compounds than that of the individual bile salt systems used before (Waters et al., 2016), confirmed by the behaviour of all compounds into binding solutes favouring the formed micelles. This developed MLC method has a higher predictive ability for HIA (*R* ^2^_PRED_ = 81%) compared with previous models. Overall, it can be concluded that there is a close resemblance between the ‘physiologically occurring’ and ‘synthetic bile salt–phospholipid micellar mixture’ used in this MLC method. This helped the compounds to behave in a manner closer to how they permeate through the human intestine, therefore simulating the human intestinal absorption process to some extent and ultimately leading to the construction of a mathematical model with a high predictive ability for HIA.
